# Radiation-Induced Fibrosarcoma in the Stoma: A Case Report and Literature Review

**DOI:** 10.1155/cris/5313214

**Published:** 2025-04-21

**Authors:** Zhao Li, Liang Fang, Liang Lv, Xinjia He, Wenqiang Luo, Dong Guo, Zhen Liu

**Affiliations:** ^1^Department of Gastrointestinal Surgery, The Affiliated Hospital of Qingdao University, Qingdao, Shandong, China; ^2^Department of Gastroenterology, The Affiliated Hospital of Qingdao University, Qingdao, Shandong, China; ^3^Department of Oncology, The Affiliated Hospital of Qingdao University, Qingdao, Shandong, China; ^4^Department of Emergency Surgery, The Affiliated Hospital of Qingdao University, Qingdao, Shandong, China

**Keywords:** colostomy, ELAPE, fibrosarcoma, radiation therapy

## Abstract

Fibrosarcoma is a rare malignant neoplasm consisting of fibroblasts with a large variety of collagen production. They usually involve deep soft tissues in extremities and trunk. However, fibrosarcoma can be seen in fields that received previous irradiation. Here, we report a case of parastomal fibrosarcoma after laparoscope-assisted extra-levator abdominal perineal resection (ELAPE) and colostomy. Prior to surgery, the patient underwent neoadjuvant chemoradiotherapy. The patient received extensive stomal lumpectomy and stoma reconstruction. The patient is free of local or distal recurrence for 1 year and died 4 years after diagnosis.

## 1. Introduction

We present a case of fibrosarcoma near stoma and review of the literature. Informed consent for the publication of the manuscript and any associated images or date was approved by the patient. Adult-type fibrosarcoma is exceedingly rare, which accounts for less than 1% of adult soft tissue sarcomas. It is most often seen in middle or older age male adults [1]. Presenting as nonspecific deep soft tissue mass, fibrosarcomas usually involve trunk, extremities, head, and neck [2]. However, in tissues that received previous irradiation therapy or foreign material implantation, fibrosarcoma can also be found [1, 3]. With the increasing accurate diagnosis of mesenchymal and nonmesenchymal tumors that mimic fibrosarcoma, the incidence of fibrosarcoma has declined drastically over the past decades [4].

Different from undifferentiated pleomorphic sarcomas, adult-type fibrosarcoma shows less than a moderate degree of pleomorphism. Microscopically, the monomorphic spindle cells are typically arranged in a uniform, fasciculated pattern. Another feature is the presence of variable amount of collagen production. Scarce, delicate intercellular collagen distribution is usually seen in poor-differentiated sarcoma. Fibrosarcoma usually expresses vimentin but is negative for S-100, desmin, and cytokeratin by immunohistochemistry [5]. Sarcomas with myofibroblastic differentiation may show SMA expression [1].

The management of fibrosarcoma depends on its location with extensive surgical excision the main therapy.

## 2. Case Presentation

A 68-year-old man was admitted in December 2018 due to difficult defecation. He denies abdominal pain, bloating, or hematochezia. He also denied any family history of rectal cancer. Colonoscopy revealed a mass 46 mm distant from anal edge from up to 10 cm involving the rectum. Pathology shows poorly differentiated adenocarcinoma. MRI shows locally advanced rectal cancer (T4bA4N2Mx). The circumferential resection margin (CRM) was positive. No distal metastasis was detected. He received neoadjuvant chemotherapy and local radiotherapy (DT5000cGy/25f) prior to curative surgery. The chemotherapy regimen is CapOX (capecitabine and oxaliplatin). The patient was well tolerated to the therapeutic doses of radiotherapy during the treatment period. He reported only mild nausea after the first course of treatment. No acute toxicity reaction such as abdominal symptoms or hematochezia was observed.

In July 2019, the patient underwent laparoscope-assisted extra-levator abdominal perineal resection (ELAPE) and colostomy. The tumor is located 11.5 cm from the upper cutting edge and 4 cm from the lower cutting edge. The tumor regression grade is 1. R0 resection was achieved. The pathology showed that the mucosa of rectum with chronic inflammation, a small amount of residual cancer was seen, the upper and lower surgical margins were negative, the rectum serosa was infiltrated, and 4/9 of lymph nodes was metastasized. He received parenteral nutrition after the operation. Liquid diet was offered 3 days after surgery at which flatus from stoma was observed. He changed to normal diet gradually and was discharged on the seventh day after surgery. No complications such as perineal wound dehiscence or discomfort were observed on 1-month follow-up. His exhaust and defecation from the stoma were normal.

**Figure 1:**
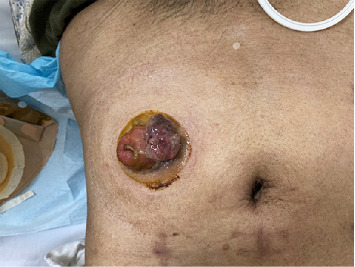
The patient received lumpectomy in an out-patient setting.

**Figure 2:**
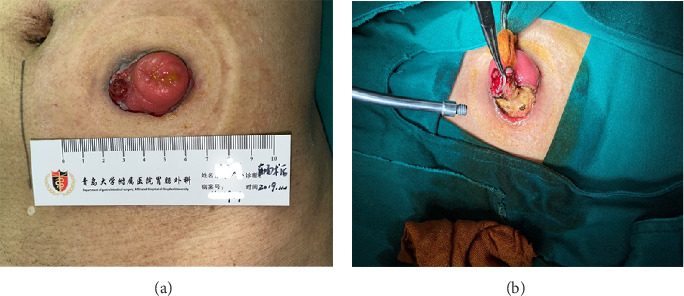
(a) A 2.5 × 1.5 cm sized mass on the stoma. (b) The neoplasm invaded colon and adjacent abdominal wall.

**Figure 3:**
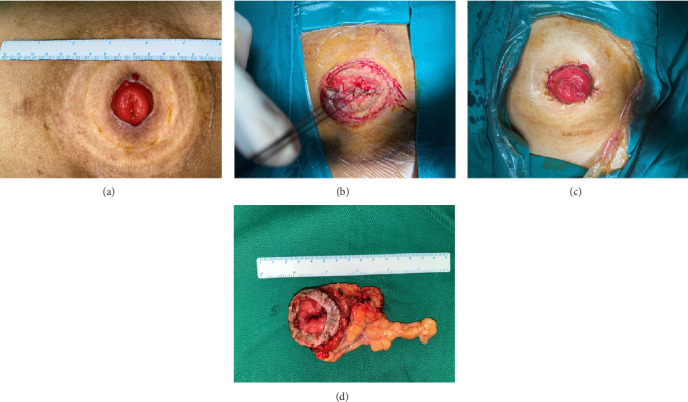
(a) Collapsed stoma prior to reconstruction. (b) The stoma under reconstruction. (c) The stoma after reconstruction. (d) Removed colon tube after reconstruction.

The patient found a 2.5 × 1.5 cm sized mass on the stoma 3 months after surgery, see Figure 1. He received lumpectomy on the same day in an outpatient setting. High-grade fibrosarcoma with Vimentin, Ki-67, and Caldesmon positive was diagnosed by immunochemistry. Extensive surgical excision was performed in November 2019, see Figure 2a,b. The patient received stoma reconstruction in April 2020, see Figure 3a–d. No remnant or local recurrence of fibrosarcoma was discovered. The patient was free of symptoms on 1-month and 6-month follow-up after the surgery. No local or distal recurrence was observed. After oncology department follow-up every 3 months, the patient died in December 2023 due to multiple vertebral metastases.

## 3. Discussion

In this case, we describe a patient with locally advanced rectal cancer who underwent initial neoadjuvant chemoradiotherapy and subsequent curative surgery and colostomy. Three months after surgery, a neoplasm occurred in the stoma site. No pain or bleeding in the stoma site was reported and the stoma functions well. Enhanced CT scan is helpful in diagnosing tumor invasion of the colon and abdominal wall. A typical “herringbone” architecture composed of fibroblasts with variable collagen production is usually seen in fibrosarcoma. With the expression of vimentin and smooth muscle actin, fibrosarcoma can represent myofibroblastic differentiation. The neoplasm was removed under extensive surgery and the stoma was reconstructed.

Neoadjuvant chemotherapy and local radiotherapy have proven to be effective in the treatment of locally advanced rectal cancer prior to curative surgery [6]. Compared to high rectal cancer, neoadjuvant chemoradiotherapy is more beneficial to low and middle rectal cancer [7]. The NCCN guidelines recommend that a radiation dose of 45–50 Gy in 25–28 fractions should be delivered in 3–4 radiation fields. After 5.5–6 weeks of treatment followed by 5–6 weeks interval, the curative is performed. Appropriate position and other measures should be taken to minimize the volume of small bowel exposure to the radiation field. The patient in our institute received 50 Gy radiation dose in 25 fractions for a treatment period of 6 weeks.

As for the concurrent chemotherapy, capecitabine was recommended by NCCN as a radiotherapy sensitizer for the treatment of rectal cancer [8]. The combination of oxaliplatin with capecitabine remains controversial [9, 10]. In our institute, patients with high-risk rectal cancer received CapOX regime as the standard therapy.

Exposure to ionizing radiation has long been recognized as a risk factor for sarcomas [11]. Radiation-induced fibrosarcoma (RIS) may occur after the radiotherapy of rectal cancer, breast cancer, or lymphoma. Case reports on RIS in patients who received colostomy are relatively few, particularly in stoma sites. RIS tends to occur at the edge of the irradiated field [12]. In the present case, the colon tube at the edge of the irradiated field became the stoma site after surgery, and the rectum in the irradiated field was removed by surgery. Radiation dose of 55 Gy or above increases the risk of sarcoma tumorigenesis [13]. It is believed that ionizing radiation can induce genomic instability [14]. P53 gene mutations contribute to the pathogenesis of RIS [15]. Postradiation sarcoma can occur decades after radiation exposure, with a median time of 10 years [16]. The patient developed fibrosarcoma 3 months after surgery and 6 months after the last course of radiotherapy. The rarity of the disease and variable time span after radiation exposure make early diagnosis difficult. For patients undergone radiation exposure, any neoplasm in the stoma site should remind the possibility of malignancy.

Adequate surgical resection with negative margins is the standard treatment for most fibrosarcoma. It is challenging to maintain stoma function after extensive surgical resection. Complications such as stoma collapse and stricture may occur. Stoma reconstruction is the treatment of choice. With a relatively high risk of recurrence, RIS tends to be aggressive with frequent local or distal metastasis. The prognosis of RIS is poor. The 5-year survival rates were reported to be less than 30% [17]. Patients diagnosed with RIS are recommended to follow-up every 3 months for stoma function evaluation. In our case, the patient died 4 years after diagnosis. Indications should be followed strictly for patients who will benefit from radiotherapy.

## 4. Conclusion

RIS in the stoma site is exceedingly rare. No guidelines were published considering the rarity of RIS. With a high risk of recurrence even after extensive resection, the prognosis is poor. Our case may raise the awareness of the need to consider unusual diagnoses of complications in the soma site and the role of the MDT approach in managing this rare clinical condition. Joint efforts with colorectal surgeon, enterostomy therapist, and radiation oncologists should be made to improve quality of life and outcome.

## Data Availability

The data that support the findings of this study are available upon request from the corresponding author. The data are not publicly available due to privacy or ethical restrictions.
